# Community-level physiological profiling analyses show potential to identify the copiotrophic bacteria present in soil environments

**DOI:** 10.1371/journal.pone.0171638

**Published:** 2017-02-07

**Authors:** Salvador Lladó, Petr Baldrian

**Affiliations:** Laboratory of Environmental Microbiology, Institute of Microbiology of the CAS, Prague, Czech Republic; Universite Paris-Sud, FRANCE

## Abstract

Community-level physiological profiling (CLPP) analyses from very diverse environments are frequently used with the aim of characterizing the metabolic versatility of whole environmental bacterial communities. While the limitations of the methodology for the characterization of whole communities are well known, we propose that CLPP combined with high-throughput sequencing and qPCR can be utilized to identify the copiotrophic, fast-growing fraction of the bacterial community of soil environments, where oligotrophic taxa are usually dominant. In the present work we have used this approach to analyze samples of litter and soil from a coniferous forest in the Czech Republic using BIOLOG GN2 plates. Monosaccharides and amino acids were utilized significantly faster than other C substrates, such as organic acids, in both litter and soil samples. Bacterial biodiversity in CLPP wells was significantly lower than in the original community, independently of the carbon source. Bacterial communities became highly enriched in taxa that typically showed low abundance in the original soil, belonging mostly to the Gammaproteobacteria and the genus *Pseudomonas*, indicating that the copiotrophic strains, favoured by the high nutrient content, are rare in forest litter and soil. In contrast, taxa abundant in the original samples were rarely found to grow at sufficient rates under the CLPP conditions. Our results show that CLPP is useful to detect copiotrophic bacteria from the soil environments and that bacterial growth is substrate specific.

## Introduction

Microbial communities in forest soil have an essential role in organic matter decomposition and thus provide critical services to the ecosystem. This role is of high importance considering the consequences of C storage or release and the fact that forest biomes currently represent a large C sink [1]. Still, utilization of various organic compounds by soil microbiota remains only partially understood or inferred from genome or metagenome sequencing [2]. Community-level physiological profiling (CLPP), based on sole C source utilization patterns, has been extensively used in the past with the aim to characterize the metabolic versatility of microbial communities in many different environments such as soils, the soil rhizosphere, wetlands and even marine environments, producing comparable data among very different environmental communities [3–6]. CLPP has also proved to be a suitable tool for the characterization of nutritional physiology of individual microorganisms [7,8]. BIOLOG plates (BIOLOG Inc, Hayward, CA, USA) are the most widely used tools for performing CLPP [9]. BIOLOG microtiter plates were originally developed for classification of bacterial isolates, primarily gram-negative (GN) species of clinical importance, based on their ability to oxidize 95 different carbon (C) sources [10]. Later, Garland & Mills [11] were the first to adapt the BIOLOG method for characterizing the functional potential of microbial communities. Both BIOLOG GN2 and ECO plates, containing 95 or 31 substrate each, have been used for this purpose [12,13]. The method is based on the redox reaction of tetrazolium violet, a redox indicator.

The advantages of CLPP over cell culture and molecular level RNA/DNA amplification-based techniques are the simplicity of the protocol and the largely reduced cost. However, many limitations in the use of this approach for complex environmental samples have been previously reported. These problems include the potential preference of fast-growing bacteria in the assay, the need to ensure equivalence of inoculum sample size, the incubation time, the data analysis and the interpretation of the CLPP results [14–19]. Preliminary results indicate that CLPP indeed selects certain taxa from the whole soil community [15], but the question about which actual taxa are enriched or reduced in the assay was never answered in detail. Considering the fact that competitive, fast-growing taxa should perform well in the assay, CLPP can be actually used to select for microorganisms with such traits that are able to utilize various C compounds and that can be subsequently identified using molecular methods.

In the present work we have used BIOLOG GN2 plates to obtain and compare the CLPP of bacteria from litter and soil of a coniferous forest dominated by *Picea abies* in the Czech Republic. The aim was to ultimately solve the question about what taxa are enriched in CLPP on various substrates and to use this method to identify competitive, copiotrophic members of the community that are able to utilize selected substrates, relevant for the studied environment. Even considering the biases mentioned above, the method should be able to positively identify those taxa that use specific C substrates for growth and that may proliferate in microniches where these substrates are available or at the time when their availability is high. Copiotrophic bacteria are key players in the soil C cycle, especially during the period of the year when vegetation is photosynthetically active and exudes large amounts of simple C compounds such as sugars, amino acids and organic acids [20]. The fact that rhizodeposition may account for 30% of total net primary production in forests [21], underlines the importance of this resource and those microorganisms that utilize it. Although often overlooked, microorganisms in C-rich root-associated niches mediate a significant part of the soil C cycle [22]. This information should complete our view of the bacterial community functioning that now considers the fact that hotspots and hot moments of activity exist in the soil [23,24].

In a previous study working with isolated strains belonging to soil abundant OTUs [7], we described that members of the *Proteobacteria* showed the widest spectrum of C source utilization and fast growth in vitro. We expect to find a significantly elevated number of *Proteobacteria* than in the original soil and litter samples, while the fraction of *Acidobacteria* that dominate the studied environment, will be reduced after CLPP. In addition, we also hypothesize that the total bacterial diversity found in the different BIOLOG wells will be substrate-dependent, with low abundance of the most dominant forest soil bacterial strains due to their strict adaptation to a low-nutrient environment. The experiment should also demonstrate which substrates are most readily utilized in the particular studied environment.

## Materials and methods

### Study site, sample collection and recovery of bacterial cells

The study area was located in the highest altitudes of the Bohemian Forest National Park, Czech Republic (Central Europe, 49°2'38"N 13°37'2"E) and was covered by an unmanaged spruce (*Picea abies*) forest. The research permit was granted by the Administration of the National Park and Protective Landscape Area of Šumava (http://www.npsumava.cz/en/3041/sekce/contacts/). The composition of total and active bacterial communities and microbial transcription in the study area have previously been explored, as have the characteristics of selected dominant bacterial taxa isolated from litter and soil [7,25,26]. Sampling was carried out in late summer (September 2014). At three sites located 100 m from each other, five topsoil samples were collected along a transect of 40 m using a soil corer with a 4.5-cm diameter. Fresh samples were transferred to the laboratory and processed within 24 h. Litter (L) horizon and organic soil (S) horizon materials from each of the three study sites were separately pooled. After the removal of roots, the L horizon material was cut into 0.5-cm pieces and mixed. The S horizon material was passed through a 5-mm sterile sieve and homogenized.

For bacterial community recovery, 5 g of litter and soil composite samples representing each site were diluted in 45 mL of 0.25% Ringer saline solution (NaCl 2.25 g L^-1^, KCl 0.105 g L^-1^, CaCl_2_ 0.045 g L^-1^ and NaHCO_3_ 0.05 g L^-1^) and agitated for 30 min. After agitation, soil and litter particles were removed by centrifugation (500 x *g* for 2 min). The bacterial fraction was recovered from the supernatant by centrifugation at 10,000 X g for 20 min. The resulting bacterial pellet was resuspended in 10 mL of sterile Ringer solution and centrifuged at 10.000 X g for 20 min; the resulting cell pellet was resuspended in 40 mL of sterile Ringer solution and used to inoculate the BIOLOG GN2 plates.

### BIOLOG GN2 assays

Twenty-five different substrates selected for being representative of soil environments were used in the study: Monosaccharides (N-acetyl-D-galactosamine, N-acetyl-D-glucosamine, L-arabinose, D-cellobiose, D-fructose, D-galactose, D-glucose, D-mannose and D-trehalose), organic acids (acetic acid, citric acid, formic acid, D-galacturonic acid, D-glucuronic acid, keto butyric acid, malonic acid and succinic acid), amino acids (L-alanine, L-asparagine, L-leucine, L-proline and L-serine) and others (uridine, thymidine and glycerol). A well with no C source was used as a negative control. A volume of 150 μL of each bacterial cell suspension in 0.25% Ringer solution was used to inoculate the wells with the selected substrates and the control well in one BIOLOG GN2 plate. Plates were then incubated for 7 days at 22°C. Spectrophotometric measurements were performed in a microplate reader at 590 nm after the inoculation and the 7 days of incubation. The absorbance value of the control well was subtracted from the absorbance in each substrate well; substrates with resulting zero or negative values were considered non-oxidized. A one-way analysis of variance (ANOVA) with Tukey’s HSD post hoc test (STATISTICA 10.0; StatSoft, Inc., Tulsa, OK, USA) was used to analyse the significance of differences in bacterial oxidation rates among substrates.

### DNA extraction and bacterial quantification

DNA was extracted from the BIOLOG GN2 wells after 1 week of incubation using a modified Miller method [27]. The length of incubation was not essential for our effort to identify the copiotrophic bacterial taxa from the forest soil but was important for the development of sufficient microbial biomass allowing robust DNA extraction. It should be noted that the length of cultivation may limit the comparability of the results of the present study with other CLPP analyses where shorter incubation times were used for samples from other environments that were less nutrient-limited. DNA was also isolated from the initial soil and litter samples and from the soil and litter diluted in Ringer’s solution before the inoculation process. The water well, containing no C source, was used as the incubated control. The number of 16S rRNA gene copies in each sample was determined with qPCR using 1108f and 1132r universal primers for bacteria [28]. For each sample, qPCR was performed in triplicate as previously described in Žifčáková et al. (2016) [25]. A one-way analysis of variance (ANOVA) with Tukey’s HSD post hoc test (STATISTICA 10.0; StatSoft, Inc., Tulsa, OK, USA) was used to analyse the significance of differences in the number of 16S copies among substrates.

### Characterization of BIOLOG GN2 bacterial communities

PCR amplification of the bacterial 16S rRNA V4 region from DNA was carried out with the barcoded primers 515F and 806R [29] using the DNA extracted as described above. Amplicons were sequenced on an Illumina MiSeq by the Argonne National Laboratory.

The amplicon sequencing data were processed with SEED 1.2.1 [30] as described in Žifčáková et al. (2016). Briefly, pair-end reads were merged using fastq-join [31]. Chimeric sequences were detected using Usearch 7.0.1090 [32] and deleted, and sequences were clustered using UPARSE implemented within Usearch [33] at a 97% similarity level. Consensus sequences were constructed for each cluster, and the closest hits at a genus or species level were identified using BLASTn against the RDP [34] and GenBank databases. Sequences identified as nonbacterial were discarded. From 16S rRNA in DNA, bacterial genome count estimates were calculated based on the 16S copy numbers in the closest available sequenced genome as described previously [35]. The Shannon Wiener index was calculated using SEED 1.2.1 [30] at 5000 bacterial sequences per sample to eliminate the effect of sampling effort. Data are available in MG RAST (4685988.3) [36].

Operational taxonomic unit (OTU) abundances in wells were calculated by multiplying the relative abundance of each OTU in each well by the number of 16S rRNA gene copies quantified by qPCR. OTU abundances were used for Principal Component Analysis (PCA). PCA was performed using the Canoco 4.5 package (Microcomputer Power, Ithaca, NY, USA) to find the relationships between bacterial growth, bacterial diversity, C sources and the origin of the sample (such as litter, soil or the site). A multivariate analysis of similarity (ANOSIM) (PAST 3.11; University of Oslo, Oslo, Norway) was used to analyse the significance of differences in the relative abundances of the 50 most abundant OTUs within substrates and samples.

Growth of individual OTUs in every S or L sample was considered significant for those substrates where the OTU abundance increased 5 times in comparison with its respective water control in at least 2 of the 3 samples from the same horizon. L-asparagine and L-serine results were not used due to missing replicates.

## Results

### Bacterial growth and substrate utilization

Oxidation of all 25 substrates was recorded after a 7-d incubation of the BIOLOG GN2 plates for most samples (Table 1). Bacterial communities from both soil and litter showed significantly higher colour development (P < 0.05) on monosaccharides (mean OD increases of 1.049 and 1.419 in litter and soil, respectively; L1 was excluded from the analysis due to outlier values) and amino acids (1.021 and 1.321) as sole sources of C than on other substrates, e.g., organic acids (0.396 and 0.504). The highest colour development among monosaccharides was recorded in the wells with D-trehalose, D-glucose, and D-fructose; the utilization of N-containing monosaccharides was slower. L-asparagine and L-proline were the most rapidly utilized amino acids (Table 1).

**Table 1:** Well colour development (OD increase) in the BIOLOG GN2 plates in the presence of bacteria extracted from the Picea abies forest litter (L) and soil (S) after 7-d incubation.

Substrates	Type of C	L1	L2	L3	S1	S2	S3
water		-0.073	0.036	-0.094	-0.018	-0.040	0.039
N-Acetyl-D-galactosamine	Carbohydrate	0.297	1.120	1.068	1.008	1.068	1.333
N-Acetyl-D-glucosamine	Carbohydrate	0.393	0.992	1.051	0.912	1.073	1.149
L-Arabinose	Carbohydrate	0.342	1.539	1.136	1.156	1.400	1.484
D-Cellobiose	Carbohydrate	0.436	1.462	0.915	1.018	1.381	1.473
D-Fructose	Carbohydrate	0.476	2.050	1.311	1.434	1.923	1.545
D-Galactose	Carbohydrate	0.382	1.607	1.172	1.307	1.817	1.600
D-Glucose	Carbohydrate	0.416	1.925	1.286	1.392	1.840	1.576
D-Mannose	Carbohydrate	0.509	1.591	0.970	1.368	1.562	1.385
D-Trehalose	Carbohydrate	0.549	1.972	1.350	1.499	1.889	1.720
Acetic Acid	Organic Acid	0.074	0.164	0.078	0.462	0.211	0.164
Citric Acid	Organic Acid	0.379	0.812	1.207	0.768	0.763	0.942
Formic Acid	Organic Acid	0.070	0.065	0.075	0.107	0.093	0.128
D-Galacturonic Acid	Organic Acid	0.213	0.946	0.934	0.912	0.958	1.007
D-Glucuronic Acid	Organic Acid	0.153	0.732	0.656	0.714	0.733	0.749
Keto Butyric Acid	Organic Acid	0.316	0.838	0.496	0.546	0.802	0.774
Malonic Acid	Organic Acid	0.189	0.422	0.306	0.215	0.403	0.222
Succinic Acid	Organic Acid	0.026	0.313	0.051	0.238	-0.005	0.199
L-Alanine	Amino Acid	0.453	1.327	0.863	1.156	1.292	1.053
L-Asparagine	Amino Acid	0.508	1.673	1.342	1.519	1.604	1.593
L-Leucine	Amino Acid	0.790	1.495	0.832	1.318	1.546	0.781
L-Proline	Amino Acid	0.522	1.939	1.350	1.461	1.918	1.699
L-Serine	Amino Acid	0.227	1.201	0.789	1.069	0.905	0.935
Uridine	Other	0.123	0.684	0.374	0.496	0.740	0.539
Thymidine	Other	0.457	0.893	0.689	0.979	0.721	0.940
Glycerol	Other	0.260	1.054	0.563	0.791	1.157	0.639

**Fig 1:**
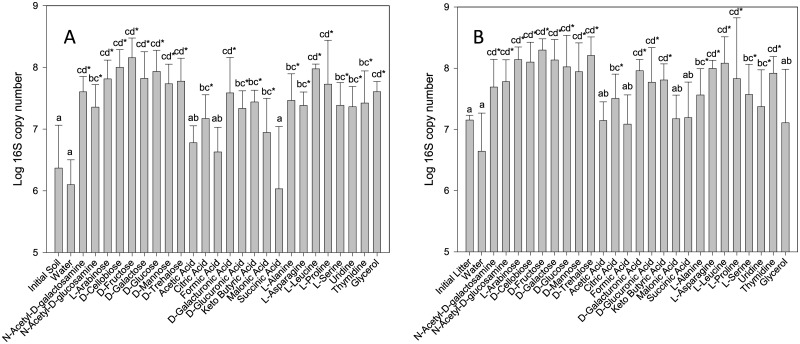
Soil (A) and litter (B) 16S rRNA gene copies in the selected BIOLOG GN2 wells after a 7-d incubation. The same lowercase letters indicate lack of a statistically significant difference (P > 0.05) between C sources. An asterisk represents the occurrence of significant differences (P < 0.05) between each C source and the water control.

The qPCR showed that there was no significant increase in the 16S copy numbers during plate incubation in the control wells with no substrate addition (“water“), while the 16S rRNA gene copy numbers in the DNA extracted from the plates were significantly higher in those wells where absorbance increased, indicating bacterial growth, than in the control wells (Fig 1). Strong correlation between the number of 16S rRNA gene copies and the colour development was found in the wells (S: R = 0.73; L: R = 0.56). Acetic acid, formic acid and succinic acid were the only three substrates not supporting significant bacterial growth in soil samples. This fact is consistent with the absorbance results, where these same three substrates presented a much lower absorbance increase than the rest. In litter samples, no significant growth was detected for acetic acid, formic acid, succinic acid, malonic acid and glycerol. When comparing bacterial growth by different type of substrates, the results were again similar between soil and litter plates, being significantly faster on carbohydrates and amino acids than on organic acids.

### Bacterial community composition after growth on different C sources

The sequences obtained by amplicon sequencing of bacterial 16S clustered into 7855 OTUs at a 97% similarity threshold after excluding global singletons. The bacterial communities were slightly but not significantly more diverse in the litter than in the soil samples both prior to bacterial extraction with Ringer saline medium (Shannon Index values of 5.78 ± 0.72 and 5.18 ± 0.41, respectively) as well as after extraction (5.78 ± 0.58 and 5.44 ± 0.25). After S and L bacterial populations were extracted, inoculated into BIOLOG GN2 plates and incubated for a 7-d period, a significant drop in biodiversity was detected, which was independent of the C source (litter 1.57 ± 0.49; soil 1.71 ± 0.58; S1 Table).

**Fig 2:**
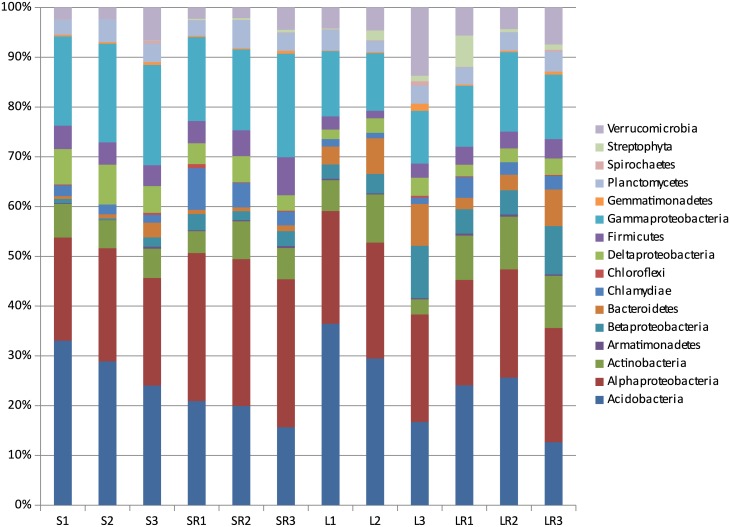
Phylogenetic composition of bacterial sequences from *Picea abies* forest. Samples: litter (L) and soil (S) prior to and after the recovery of bacterial communities with a saline medium (LR and SR). The data represent mean values for the three composite samples and express the estimated relative abundances of bacterial genomes.

Bacterial sequences from soil and litter, before inoculation into BIOLOG plates, belonged to 16 phyla, but only 10 were recovered with abundances over 0.5% (Fig 2) in at least one sample. In both horizons, *Acidobacteria* and Alphaproteobacteria were dominant, comprising 65–80% of all bacteria. The most abundant OTUs in S and L communities belonged to the Acidobacterial genus *Granulicella* (S: 16.7%; L: 11.6%). Other abundant Acidobacterial genera were *Acidobacterium* (S: 4.6%; L: 6.7%), Candidatus *Solibacter* (S: 5.0%; L: 5.1%) and *Telmatobacter* (S: 1.3%; L: 2.6%). Other abundant genera included the Actinobacteria *Mycobacterium* (S: 1.5%; L: 0.3%) and *Conexibacter* (S: 0.6%; L: 1.0%) and the Proteobacteria *Beggiatoa* (S: 4.4%; L: 0.6%), *Bradyrhizobium* (S: 3.0%; L: 3.7%), *Desulfomonile* (S: 5.5%; L: 1.0%), *Methylocystis* (S: 3.5%; L: 3.3%), *Pseudomonas* (S: 3.2%; L: 2.9%), *Rhodoplanes* (S: 8.7%; L: 5.3%) and *Thioprofundum* (S: 4.6%; L: 1.2%). Several taxa showed preferential associations with either litter or soil (S2 Table). Bacterial communities present in the Ringer extracts were highly similar in terms of diversity to those detected in the initial samples (Fig 2; S2 Table). However, members of the phylum *Acidobacteria* were slightly underrepresented.

After the 7-d incubation of S and L extracts with the C substrates, the same ten phyla were recovered with abundances over 0.5% (Fig 2) in at least one sample. *Proteobacteria* and *Bacteroidetes* were dominant after incubation, comprising almost 100% of all bacteria in the vast majority of samples (S3 Table). Bacterial communities after incubation typically showed low evenness due to a remarkable rise in the relative abundance of a few of the most abundant OTUs. The 10 most abundant OTUs (found in more wells at a relative abundance >0.5% in each of them) accounted for more than 70% of all genomes in 94 wells, 80% in 77 wells and 90% in 43 wells, from a total of 153 analysed wells. Of these 10 OTUs, nine belonged to the Proteobacteria and four to the genus *Pseudomonas* (S3 Table).

**Fig 3:**
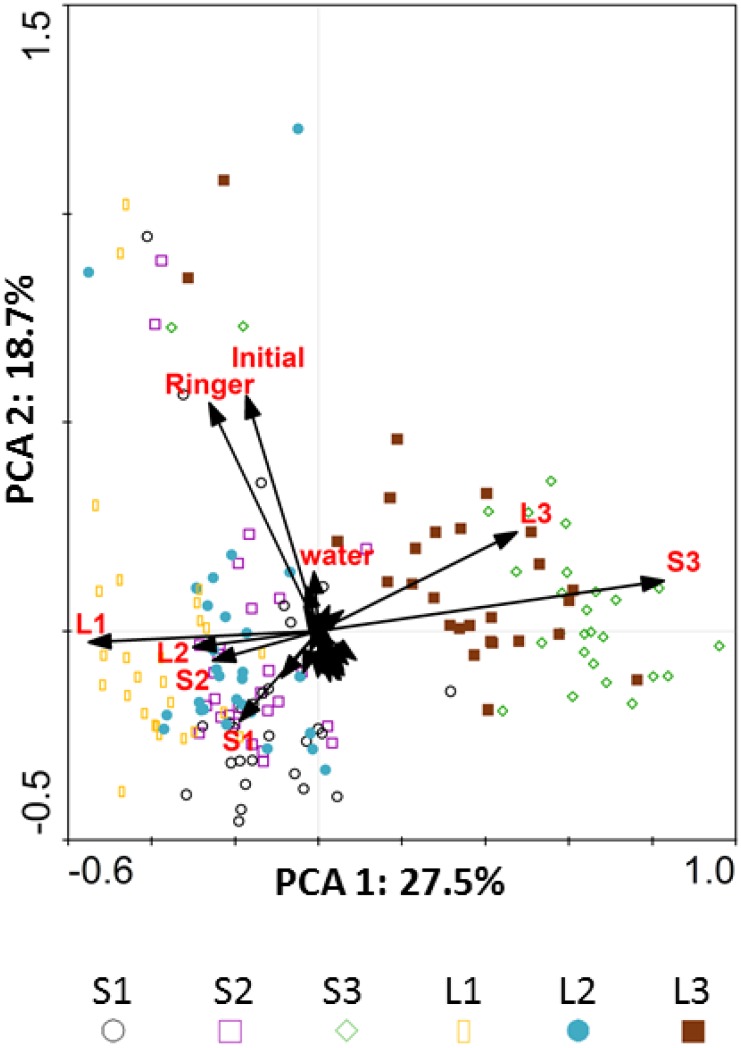
Principal Component Analysis (PCA). Biplot showing scores (bacterial biodiversity in each BIOLOG GN2 well analysed in the study) and environmental variable vectors (Samples: S or L; Sites: 1, 2 and 3; Controls: Initial soil prior to bacterial community recovery, after Ringer recovery and water wells in the BIOLOG GN2 system; Less weighted environmental vectors, without legend in the graph, correspond to the different Biolog substrates).

Bacterial community composition in the initial substrate and in the corresponding Ringer extract were rather similar according to the PCA analysis but were different from the communities in the wells after incubation (Fig 3), which is due to the selective increase of certain taxa in most substrate-containing wells. The bacterial community in control wells with water also underwent development during incubation as indicated by the fact that they were more similar to wells with substrates after incubation than to the original communities.

**Fig 4:**
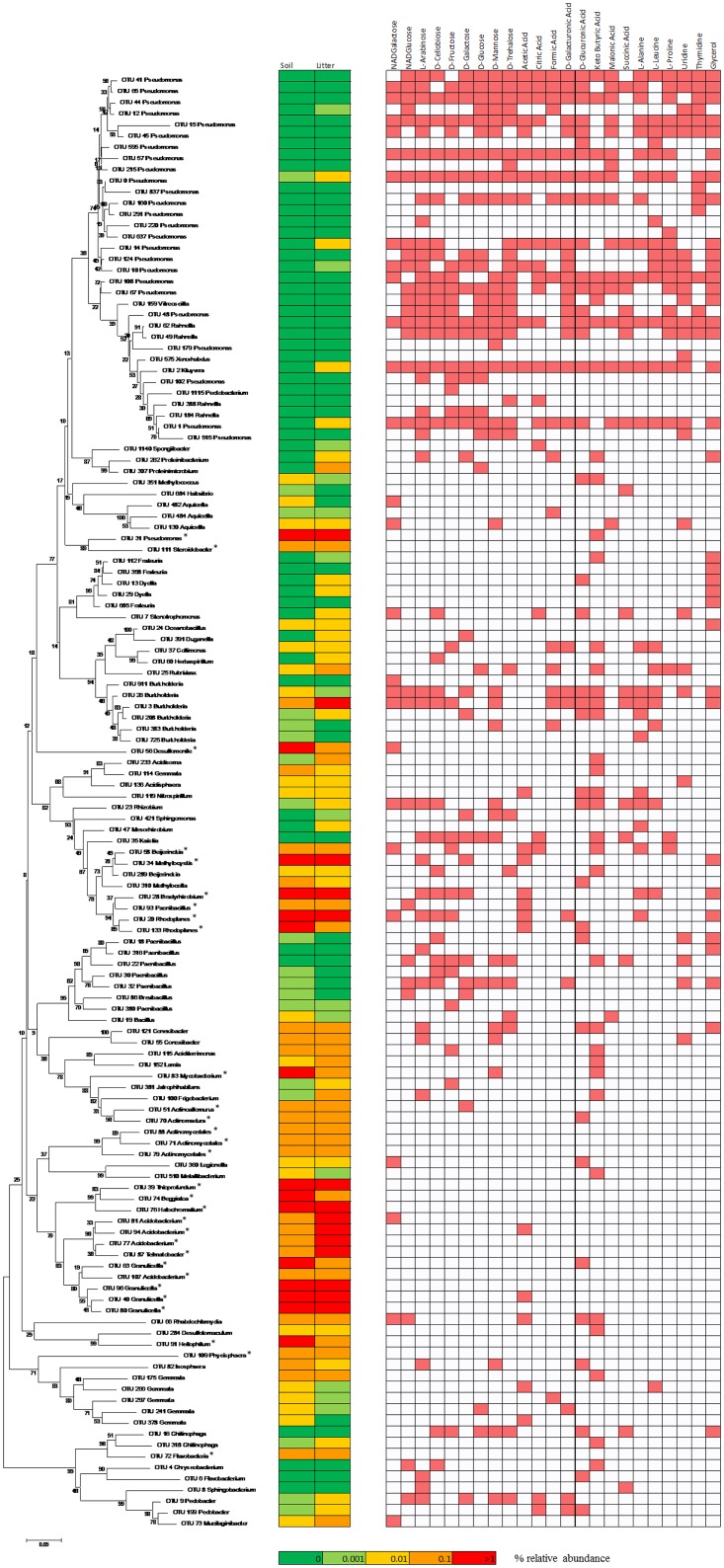
Neighbour-joining phylogenetic tree with individual OTU growth by substrate. Based on bacterial OTUs (16S rRNA gene) from the *Picea abies* forest soil with detectable growth on different C sources and their relative abundances in soil and litter (on a logarithmic scale). Asterisks highlight OTUs belonging to the 30 most abundant OTUs in the forest soil. Growth is indicated as positive (red background) if the abundance of the OTU increased at least 5-fold in at least two of the three wells with a given substrate in either soil or litter samples.

In soil and litter samples, 115 OTUs were detected to grow on at least one substrate as indicated by the fact that their 16S copy numbers increased at least 5-fold compared to water. These 115 OTUs belonged to 58 different bacterial genera. Of the OTUs, 66% belonged to the phylum *Proteobacteria* and just 3% to the dominant soil phylum *Acidobacteria*. OTU 0 and OTU 44, both belonging to the genus *Pseudomonas*, were able to grow with all 23 tested substrates indicating their catabolic versatility. Members of the *Pseudomonas* and *Rahnella* genera (Gammaproteobacteria), *Isosphaera* (Planctomycetes), *Rubrivivax* (Betaproteobacteria) and *Paenibacillus* (Firmicutes) grew on 20 or more substrates (S4 Table). The growth of most OTUs was supported by L-arabinose (41), keto-butyric acid (39), D-glucuronic acid (38 OTUs) and glycerol (36) (S4 Table). Of the 10 OTUs able to grow using more than 20 different substrates, none was found in a relative abundance over 0.1% in the original soil or litter samples (Fig 4). Furthermore, just one single OTU of those that were able to significantly grow on more than 10 substrates, OTU 3 belonging to the genus *Burkholderia*, was present at a relative abundance above 0.1% in the original samples (Fig 4). Among the 30 most abundant OTUs in the forest topsoil, 15 were detected as growing rapidly on at least one substrate, and four of them grew in more than 2 substrates: OTU 58 (*Beijerinckia*, 4 substrates), OTU 34 (*Methylocystis*, 7 substrates), OTU 28 (*Bradyrhizobium*, 8 substrates) and OTU 20 (*Rhodoplanes*, 8 substrates) (Fig 4).

## Discussion

In the present work, we have used qPCR and Illumina sequencing to identify the trends in bacterial community composition development in CLPP using the BIOLOG GN2 plates to study a forest soil environment. Although limitations of this method as a technique for CLPP have been previously shown by [15], only the use of current molecular methods allowed us now to ultimately demonstrate the composition of the bacterial community after the assay.

Our results show that only a specific and limited subset of the total forest soil bacterial community contributes to substrate utilization in the BIOLOG GN2 wells, although it is obvious that the diversity of taxa utilizing some of the tested compounds, such as glucose for example, must be very high. Moreover, the taxa that become most enriched in the assay and are thus responsible for the bulk of the metabolic response (substrate-induced respiration) mostly show low abundance in the original soil as well as in the community of bacteria extracted with Ringer solution. The fact that the dominant bacteria from the site of this study also demonstrated their ability to grow on the tested substrates in BIOLOG GN2 plates when tested as single cultures [7] indicates, that their absence in the present study is either due to slow growth or the inability to compete with others. Taken together, communities of bacteria after CLPP on BIOLOG GN2 plates become highly enriched in particular bacterial OTUs; those that grow at a sufficient rate and are competitive under the conditions of the study, i.e. at high concentrations of C sources. Those OTUs with the highest enrichment in the BIOLOG GN2 plates belonged to *Proteobacteria*, especially to the class Gammaproteobacteria and the genus *Pseudomonas*. Members of the Gammaproteobacteria have been repeatedly described as r-strategists, dominating nutrient-rich habitats such as the rhizosphere [37–39]. The BIOLOG GN2 system seems to not be suitable for the metabolic characterization of communities from forests developed on acidic soils, where the nutrient abundance is typically low and the limited amount of available substrates leads to the selection of slow growing bacteria with more efficient metabolisms. In such environments, slow growing oligotrophs (K-strategists), such as the members of the phylum *Acidobacteria*, are often dominant [25,40,41] and significantly contribute to microbial activity [26]. Since the environmental abundance of the r-strategists that were enriched on the BIOLOG GN2 plates in this study is low, the results indicate that the content of easily accessible C sources in the studied environment is either low or constricted to rare microhabitats. It has been proposed that spatial heterogeneity may explain the outstanding bacterial diversity in soil environments [42–44]. The fact that soil microhabitats can widely differ in their nutrient content or physical properties may affect the bacterial community composition and allow the existence of less competitive strains.

The study by [15] showed that different DGGE bands became dominant in BIOLOG plates in wells with different substrates, showing a substrate-dependent community shift in the original populations extracted from the soil rhizosphere. Unfortunately, DGGE has a very low resolution for complex soil communities, hindering further conclusions. In the present study, we were able to demonstrate by qPCR of the 16S rRNA gene that there is indeed significant substrate-dependent bacterial growth. The significantly higher number of 16S rRNA copies in wells containing carbohydrates and amino acids instead of organic acids for both soil and litter communities confirms that different substrate categories support growth at different rates in this particular environment. This observation was in agreement with the strong correlation between the number of 16S rRNA gene copies and the colour development in the wells. However, the communities after incubation grouped in the PCA according to sample rather than according to substrate. Although the effect of substrate on bacterial community composition might still be significant, this fact highlights the influence of the original composition of bacterial communities in soil and litter samples on the community composition after cultivation. In addition, the multivariate statistical analysis revealed that the bacterial diversity present in the wells with carbohydrates, amino acids or organic acids was significantly different within substrate categories in terms of relative and absolute abundances of the dominant OTUs, confirming our hypothesis of substrate dependent shifts in bacterial communities. However, when changing from a general view of the total bacterial diversity to a deeper look to the particular OTUs that were able to significantly increase their biomass in the different BIOLOG GN2 wells, our results show that mostly belonged to the same taxonomic groups, independently of the C substrate. For example, 21 of the 27 OTUs able to significantly grow on D-glucose belonged to the Gammaproteobacteria, the vast majority of them to the genus *Pseudomonas*. Very similar percentages were found for the other substrates, independently of the substrate category within carbohydrates, amino acids or organic acids, confirming a highly versatile metabolism for the members of this class, as described before with isolated soil strains from the same environment [7]. We also detected two and three OTUs belonging to the phylum *Acidobacteria* and the class Alphaproteobacteria, respectively, that exhibited sufficient growth in some of the wells, but these never reached percentages of abundance high enough to be considered dominant in the bacterial communities and exhibit a much less versatile metabolism than the members of the Gamma- and Betaproteobacteria.

Just 50% of the most abundant OTUs in soil and litter were detected growing in at least one substrate. In a community dominated by members of the phylum *Acidobacteria*, four of the most abundant Acidobacterial OTUs were detected. However, all of them barely overcame the growth threshold for one C source, even though we have previously shown that soil *Acidobacteria* isolates are able to grow on a wide range of low-mass carbohydrates [7] and in other compounds such as amino acids and organic acids to a lower extent [45,46]. As aforementioned, the *Acidobacteria* were slightly underrepresented after the Ringer recovery, when compared to the initial samples. This fact could suggest that the bacterial extraction method from soil and litter samples was negatively affecting the Acidobacterial populations prior to the inoculation of the Biolog plates. However, the high similitude, in terms of bacterial diversity, between the initial samples and immediately after the bacterial recovery with Ringer, shows that the slightly underrepresentation is not significant to justify the highly remarkable decrease in abundance of the members of the *Acidobacteria* in the Biolog plates, after the incubation period. Consequently, we can note that members of the phylum *Acidobacteria* were clearly outcompeted by fast-growing bacteria in the BIOLOG wells. Among the most abundant soil and litter OTUs, the four that were detected growing in more than 2 different substrates all belonged to the order *Rhizobiales* within the Alphaproteobacteria. These four OTUs grew with between four and eight substrates, a similar profile to that of *Rhizobiales* previously isolated from the same environment [7]. Interestingly, members of the *Rhizobiales* have been very recently described as copiotrophs in an SIP experiment in microcosms [47] and this may be the reason for the detection of these bacteria in the widest range of substrates in the BIOLOG plates rather than other abundant and important members of the *Picea abies* forest soil bacterial community.

## Conclusions

CLPP using the BIOLOG system appears to be highly relevant for the screening of opportunistic r-strategists capable of fast growth that are competitive in mixed cultures, conditions to be expected at certain microniches, such as the tree root surfaces, which exude organic acids. This group of opportunistic r-strategists was in this study represented mainly by Gamma- and Betaproteobacteria. In addition, our results confirm previous studies in that they show the serious limitations that prevent the applicability of CLPP for the profiling of the metabolic versatility of whole soil communities and clearly show that such analyses may be extremely biased towards certain functional guilds of bacteria. This is most likely because the C source concentration immensely exceeds the natural concentrations.

## Supporting information

S1 Table Diversity of bacterial communities expressed as the Shannon-Wiener index after 7-d incubation of various C sources in the BIOLOG GN2 inoculated with bacteria extracted from the *Picea abies* forest litter and soil. (DOCX)

S2 Table Identification of the most abundant bacterial OTUs (OTUs > 0.5% relative abundance in at least one site) in the *Picea abies* forest litter (L) and soil (S) and in the corresponding Ringer extracts (RL and RS). Abundance data represent means and standard deviations from three samples. (DOCX)

S3 Table Relative abundance of bacterial OTUs from the Picea abies forest after incubation for 7 days with various C substrates. Data are displayed as ‰, L—litter, S—soil. (XLSX)

S4 Table Bacterial OTUs from the Picea abies forest soil with detectable growth on different C sources. Growth on given substrate is indicated as positive if the abundance of the OTU increased at least 5-fold in at least two of the three wells with a given substrate. (XLSX)
