# Supplementary material for: Community-level physiological profiling analyses show potential to identify the copiotrophic bacteria present in soil environments
Source: PLoS One. 2017 Feb 7;12(2):e0171638. doi: 10.1371/journal.pone.0171638 (PMC5295708; doi:10.1371/journal.pone.0171638)
Supplement: S1 Table — (DOCX) [file pone.0171638.s001.docx]

**Table S1.** Diversity of bacterial communities expressed as the Shannon-Wiener index after 7-d incubation of various C sources in the BIOLOG GN2 inoculated with bacteria extracted from the *Picea abies* forest litter and soil.

| **Substrates** | **L1** | **L2** | **L3** | **S1** | **S2** | **S3** |
| --- | --- | --- | --- | --- | --- | --- |
| water | 2.61 | 2.30 | 2.79 | 2.11 | 1.86 | 2.07 |
| N-Acetyl-D-galactosamine | 1.41 | 1.25 | 2.30 | 2.26 | 1.15 | 2.23 |
| N-Acetyl-D-glucosamine | 1.70 | 1.32 | 2.17 | 2.33 | 1.25 | 2.15 |
| L-Arabinose | 1.53 | 1.33 | 2.18 | 1.40 | 1.07 | 2.09 |
| D-Cellobiose | 1.29 | 0.94 | 1.94 | 1.14 | 0.73 | 1.50 |
| D-Fructose | 1.29 | 0.94 | 2.28 | 1.16 | 0.76 | 1.71 |
| D-Galactose | 1.47 | 1.24 | 2.12 | 1.79 | 1.70 | 1.99 |
| D-Glucose | 1.17 | 0.81 | 2.33 | 1.81 | 0.71 | 1.97 |
| D-Mannose | 1.48 | 1.45 | 2.19 | 1.49 | 1.16 | 1.79 |
| D-Trehalose | 1.59 | 1.47 | 2.33 | 1.49 | 1.36 | 2.09 |
| Acetic Acid | 1.63 | 1.02 | 1.93 | 2.62 | 1.20 | 1.97 |
| Citric Acid | 1.84 | 1.07 | 2.15 | 2.43 | 1.25 | 1.83 |
| Formic Acid | 2.28 | 1.35 | 2.50 | 1.57 | 1.27 | 2.36 |
| D-Galacturonic Acid | 1.50 | 1.10 | 2.23 | 2.46 | 1.66 | 2.14 |
| D-Glucuronic Acid | 0.82 | 1.51 | 2.39 | 2.76 | 1.10 | 2.30 |
| Keto Butyric Acid | 1.51 | 1.36 | 2.53 | 1.29 | 0.98 | 2.21 |
| Malonic Acid | 1.05 | 1.28 | 1.98 | 2.61 | 1.05 | 1.93 |
| Succinic Acid | 1.58 | 0.98 | 2.03 | 2.46 | 2.63 | 2.19 |
| L-Alanine | 1.61 | 1.36 | 1.66 | 2.68 | 0.96 | 1.70 |
| L-Asparagine | 1.01 | 0.95 | - | 2.88 | 0.83 | - |
| L-Leucine | 0.61 | 1.05 | 1.42 | 2.86 | 0.70 | 1.23 |
| L-Proline | 0.99 | 0.92 | 1.35 | 1.47 | 0.94 | 1.71 |
| L-Serine | - | 1.13 | 1.59 | 1.99 | 0.84 | 1.82 |
| Uridine | 2.06 | 1.59 | 2.45 | 1.73 | 1.33 | 2.21 |
| Thymidine | 1.48 | 0.99 | 2.21 | 1.31 | 1.10 | 1.94 |
| Glycerol | 1.75 | 1.41 | 1.93 | 1.88 | 1.55 | 2.22 |
