# Supplementary material for: Community-level physiological profiling analyses show potential to identify the copiotrophic bacteria present in soil environments
Source: PLoS One. 2017 Feb 7;12(2):e0171638. doi: 10.1371/journal.pone.0171638 (PMC5295708; doi:10.1371/journal.pone.0171638)
Supplement: S2 Table — (DOCX) [file pone.0171638.s002.docx]

**Table S2.** Identification of the most abundant bacterial OTUs (OTUs > 0.5% relative abundance in at least one site) in the *Picea abies* forest litter (L) and soil (S) and in the corresponding Ringer extracts (RL and RS). Abundance data represent means and standard deviations from three samples.

| **OTU** | **Best Identified hit in GenBank** | **Phylum** | **Similarity (%)** | **Relative abundance (‰)** | | | | | |  |
| --- | --- | --- | --- | --- | --- | --- | --- | --- | --- | --- |
|  |  |  |  | **DNA S** | **DNAL** | **DNA RS** |  | **DNA RL** |  | |
| 34 | *Methylocystis* sp (DQ852351) | Proteobacteria | 95.7 | 33.7±4.3 | 27.6±18.3 | 55.0±12.7 |  | 32.3±8.9 |  | |
| 20 | *Rhodoplanes* sp. (HE962154) | Proteobacteria | 98.4 | 68.6±12.1 | 31.7±20.8 | 76.1±6.6 |  | 29.4±13.0 |  | |
| 28 | *Bradyrhizobium* sp. (HQ694740) | Proteobacteria | 100.0 | 28.1±2.6 | 34.4±3.7 | 33.6±7.4 |  | 35.5±11.4 |  | |
| 31 | *Pseudomonas* sp. (AB819482) | Proteobacteria | 94.1 | 30.4±16.3 | 26.8±10.2 | 37.6±13.1 |  | 27.8±8.1 |  | |
| 39 | *Thioprofundum* sp. (AB266389) | Proteobacteria | 86.6 | 45.5±15.9 | 12.0±3.5 | 15.4±4.8 |  | 10.2±5.0 |  | |
| 40 | *Granulicella* sp. (HQ687090) | Acidobacteria | 91.9 | 75.7±41.7 | 30.3±16.5 | 8.2±1.9 |  | 11.7±2.0 |  | |
| 76 | *Halochromatium* sp. (FN293082) | Proteobacteria | 85.4 | 23.8±4.8 | 13.7±7.6 | 28.9±19.0 |  | 21.1±14.3 |  | |
| 96 | *Granulicella* sp. (HQ687087) | Acidobacteria | 90.7 | 14.1±3.7 | 13.4±4.8 | 12.3±1.8 |  | 9.1±6.1 |  | |
| 88 | *Actinomycetales* sp. (X68466) | Acidobacteria | 94.9 | 7.7±3.5 | 6.8±1.6 | 13.3±1.6 |  | 6.3±1.7 |  | |
| 81 | *Acidobacterium* sp. (AM086241) | Acidobacteria | 98.0 | 7.9±3.7 | 19.0±8.5 | 6.1±1.2 |  | 5.7±4.1 |  | |
| 93 | *Paenibacillus sp*. (EU571199) | Firmicutes | 98.0 | 8.6±4.0 | 5.4±2.2 | 14.5±1.6 |  | 13.6±7.3 |  | |
| 71 | *Actinomycetales* sp. (X68468) | Acidobacteria | 93.7 | 8.3±3.3 | 7.3±4.6 | 6.9±1.0 |  | 4.7±2.8 |  | |
| 63 | *Granulicella* sp. (HQ687090) | Acidobacteria | 92.9 | 35.0±15.4 | 6.9±2.1 | 18.9±4.0 |  | 6.1±4.0 |  | |
| 80 | *Granulicella* sp. (HQ687090) | Acidobacteria | 91.9 | 13.5±4.9 | 15.8±13.9 | 11.0±5.9 |  | 9.4±1.2 |  | |
| 107 | *Acidobacterium* sp. (AB298536) | Acidobacteria | 93.5 | 9.4±6.6 | 7.2±4.5 | 5.3±2.5 |  | 8.0±6.3 |  | |
| 77 | *Acidobacterium* sp. (AB669481) | Acidobacteria | 97.2 | 6.4±2.6 | 14.7±11.5 | 9.7±1.9 |  | 10.4±7.9 |  | |
| 87 | *Telmatobacter* sp. (KC954751) | Acidobacteria | 97.6 | 7.4±2.2 | 14.5±11.7 | 9.0±1.9 |  | 7.8±4.5 |  | |
| 111 | *Steroidobacter* sp. (JC2953) | Proteobacteria | 96.0 | 5.8±3.6 | 7.0±1.7 | 14.3±5.5 |  | 28.5±3.1 |  | |
| 91 | *Heliophilum* sp. (HF549116) | Firmicutes | 86.7 | 13.0±10.8 | 4.9±4.7 | 7.9±1.9 |  | 13.0±5.8 |  | |
| 94 | *Acidobacterium* sp. (AM086241) | Acidobacteria | 96.0 | 6.1±1.8 | 10.9±6.9 | 10.1±3.9 |  | 16.8±5.0 |  | |
| 56 | *Desulfomonile* sp. (CP003360) | Proteobacteria | 88.5 | 39.9±13.4 | 4.3±1.5 | 9.1±7.1 |  | 10.8±6.5 |  | |
| 79 | *Actinomycetales* sp. (X68467) | Acidobacteria | 92.9 | 7.5±3.3 | 6.0±2.9 | 4.3±1.3 |  | 9.6±3.9 |  | |
| 74 | *Beggiatoa* sp. (JN674459) | Proteobacteria | 84.3 | 29.0±13.7 | 2.7±2.3 | 3.9±2.2 |  | 6.2±4.1 |  | |
| 133 | *Rhodoplanes* sp. (GQ369128) | Proteobacteria | 97.2 | 11.7±7.4 | 2.7±2.4 | 27.6±25.4 |  | 12.8±4.7 |  | |
| 109 | *Phycisphaera* sp. (AP012338) | Planctomycetes | 80.2 | 7.2±3.7 | 7.2±7.4 | 16.6±9.5 |  | 3.1±1.6 |  | |
| 83 | *Mycobacterium* sp. (AB370111) | Actinobacteria | 100.0 | 14.2±10.5 | 2.2±2.0 | 7.0±3.1 |  | 3.7±2.1 |  | |
| 70 | *Actinomadura* sp. (JN413468) | Actinobacteria | 95.3 | 4.1±2.1 | 8.3±7.6 | 17.7±10.3 |  | 2.8±0.8 |  | |
| 58 | *Beijerinckia* sp. (AB680128) | Proteobacteria | 97.2 | 4.9±0.7 | 6.2±4.7 | 7.8±1.3 |  | 1.1±0.4 |  | |
| 72 | *Flavobacteria* sp. (AF491884) | Bacteroidetes | 98.4 | 3.6±3.1 | 6.1±4.6 | 5.0±1.8 |  | 8.9±5.4 |  | |
| 51 | *Actinoallomurus* sp. (AB668307) | Actinobacteria | 96.4 | 5.0±0.1 | 4.0±3.2 | 1.8±0.1 |  | 3.0±1.5 |  | |
| 180 | *Ruminococcus* sp. (GU999991) | Firmicutes | 83.2 | 9.0±5.2 | 2.5±2.5 | 9.4±4.4 |  | 5.5±0.6 |  | |
| 3 | *Burkholderia* sp. (AM268227) | Proteobacteria | 100.0 | 2.8±1.5 | 13.3±9.0 | 5.8±2.4 |  | 4.8±2.3 |  | |
| 55 | *Conexibacter* sp. (CP001854) | Actinobacteria | 95.3 | 2.0±1.1 | 4.7±3.3 | 5.4±2.5 |  | 8.9±1.5 |  | |
| 97 | *Rhizomicrobium* sp. (AB081581) | Proteobacteria | 92.5 | 2.7±0.2 | 6.1±2.0 | 2.1±0.3 |  | 4.6±3.2 |  | |
| 98 | *Granulicella* sp. (HF568987) | Acidobacteria | 98.4 | 2.3±1.6 | 12.9±9.2 | 3.1±1.1 |  | 14.5±7.5 |  | |
| 142 | *Opitutus* sp. (GQ902879) | Verrucomicrobia | 94.9 | 1.5±1.0 | 5.0±3.1 | 1.8±0.4 |  | 4.1±2.7 |  | |
| 73 | *Mucilaginibacter* sp. (EU423302) | Bacteroidetes | 99.6 | 0.3±0.1 | 5.3±1.7 | 3.2±0.7 |  | 8.7±3.3 |  | |
| 61 | *Dioscorea* sp. (EF380353) | Streptophyta | 99.6 | 0.08±0.1 | 9.3±7.6 | 4.9±2.5 |  | 0.7±0.9 |  | |
| 145 | *Steroidobacter* sp. (EF605262) | Proteobacteria | 86.3 | 1.8±0.7 | 6.9±4.5 | 0.4±0.4 |  | 6.8±2.4 |  | |
| 156 | *Granulicella* sp. (HQ687090) | Acidobacteria | 91.5 | 8.9±6.6 | 0.6±0.6 | 3.2±1.5 |  | 20.8±26.0 |  | |
| 134 | *Chthoniobacter* sp. (AY388649) | Verrucomicrobia | 92.9 | 4.4±6.9 | 13.6±23.4 | 1.2±0.7 |  | 3.2±2.3 |  | |
| 139 | *Actinoallomurus* sp. (AB668306) | Actinobacteria | 87.1 | 8.7±10.0 | 2.0±0.7 | 0.7±0.2 |  | 5.6±4.5 |  | |
| 206 | *Telmatobacter* sp. (AM887760) | Acidobacteria | 98.0 | 1.5±0.7 | 5.5±3.8 | 4.7±4.1 |  | 0.8±0.4 |  | |
| 178 | *Methylocystis* sp. (AB636303) | Proteobacteria | 97.2 | 1.4±0.4 | 4.6±3.8 | 5.5±8.7 |  | 3.6±6.3 |  | |
| 170 | *Granulicella* sp. (HQ687087) | Acidobacteria | 100.0 | 0.7±0.4 | 7.0±4.3 | 0.7±0.7 |  | 9.6±7.1 |  | |
| 231 | *Beggiatoa* sp. (JN674459) | Proteobacteria | 86.3 | 6.8±3.7 | 0.4±0.7 | 5.3±2.2 |  | 2.2±0.9 |  | |
| 209 | *Granulicella* sp. (AM887759) | Acidobacteria | 97.2 | 0.5±0.1 | 5.4±4.1 | 8.3±6.9 |  | 2.8±2.1 |  | |
| 132 | *Thiotaurens* sp. (JN882289) | Proteobacteria | 85.9 | 5.0±1.8 | 2.3±1.9 | 3.3±1.4 |  | 3.2±2.0 |  | |
| 147 | *Steroidobacte*r sp. (JC2953) | Proteobacteria | 96.4 | 1.5±1.4 | 4.5±2.9 | 1.1±0.5 |  | 4.1±3.3 |  | |
| 166 | *Candidatus Xiphinematobacter* sp. (AF217461) | Verrucomicrobia | 87.6 | 4.8±2.0 | 3.6±1.9 | 1.2±0.9 |  | 6.8±3.1 |  | |
| 347 | *Spirochaeta* sp. (JN713484) | Spirochaetes | 87.3 | 1.8±2.7 | 3.2±4.1 | 0.9±0.2 |  | 4.7±2.6 |  | |
| 66 | *Candidatus Rhabdochlamydia* sp. (HF933203) | Chlamydiae | 99.6 | 3.8±1.3 | 2.8±1.5 | 3.3±1.9 |  | 0.8±0.3 |  | |
| 140 | *Phenylobacterium* sp. (JX469404) | Proteobacteria | 96.0 | 2.6±1.7 | 4.0±2.1 | 2.9±2.7 |  | 2.6±3.8 |  | |
| 121 | *Conexibacter* sp. (CP001854) | Actinobacteria | 98.4 | 1.9±0.4 | 2.6±2.7 | 0.8±0.6 |  | 4.3±2.1 |  | |
| 52 | *Dyella* sp. (AM086248) | Proteobacteria | 100.0 | 0.07±0.06 | 2.7±3.2 | 2.3±3.1 |  | 5.9±9.7 |  | |
| 89 | *Rhodopila* sp. (AM161158) | Proteobacteria | 97.6 | 3.2±1.5 | 2.4±0.08 | 0.2±0.4 |  | 4.9±4.8 |  | |
| 168 | *Granulicella* sp. (HQ687090) | Acidobacteria | 99.6 | 0.4±0.4 | 3.6±3.2 | 0.2±0.3 |  | 6.6±9.1 |  | |
| 192 | *Rhodoplanes* sp. (HE962157) | Proteobacteria | 97.6 | 2.6±1.6 | 6.2±10.0 | 0.1±0.1 |  | 4.6±3.5 |  | |
| 154 | *Rhodoplanes* sp. (GQ369128) | Proteobacteria | 99.2 | 1.8±2.5 | 10.7±18.4 | 2.2±0.8 |  | 1.9±1.7 |  | |
| 25 | *Rubrivivax* sp. (AM086242) | Proteobacteria | 100.0 | 0.1±0.2 | 6.9±8.2 | 4.6±6.5 |  | 0.4±0.2 |  | |
| 100 | *Frigoribacterium* sp. (JQ977229) | Actinobacteria | 100.0 | 0.03±0.05 | 4.9±4.9 | 2.1±0.9 |  | 4.7±3.0 |  | |
| 185 | *Simkania* sp. (FR872582) | Chlamydiae | 90.1 | 3.2±3.3 | 0.5±0.09 | 1.8±0.8 |  | 3.3±2.1 |  | |
| 221 | *Telmatobacter* sp. (KC954751) | Acidobacteria | 97.6 | 2.0±1.7 | 3.3±2.6 | 2.8±2.5 |  | 3.1±1.6 |  | |
| 285 | *Cellulosilyticum* sp. (EF382648) | Firmicutes | 86.1 | 4.5±5.0 | 0.1±0.1 | 10.7±7.6 |  | 0.7±0.7 |  | |
| 317 | *Acidobacterium* sp. (AB298536) | Acidobacteria | 98.0 | 2.0±1.5 | 2.4±4.0 | 4.0±4.9 |  | 0.4±0.7 |  | |
| 319 | *Reyranella* sp. (JX260424) | Proteobacteria | 98.8 | 0.2±0.4 | 2.6±4.2 | 2.0±2.7 |  | 1.4±1.6 |  | |
| 195 | *Phenylobacterium* sp. (JX949351) | Proteobacteria | 98.0 | 1.7±0.6 | 3.9±2.0 | 1.4±2.2 |  | 2.2±2.9 |  | |
| 138 | *Actinomycetales* sp. (X68467) | Acidobacteria | 96.4 | 3.5±0.7 | 3.6±1.3 | 0.2±0.3 |  | 1.3±1.6 |  | |
| 189 | *Actinomycetales* sp. (X68466) | Acidobacteria | 93.7 | 2.4±1.0 | 2.5±2.4 | 0.2±0.3 |  | 2.7±4.6 |  | |
| 202 | *Desulfomonile* sp. (CP003360) | Proteobacteria | 89.3 | 6.3±4.2 | 1.9±0.6 | 0.1±0.2 |  | 1.8±3.2 |  | |
| 101 | *Rhizomicrobium* sp. | Proteobacteria | 92.5 | 0.9±0.1 | 3.0±1.8 | 5.7±9.7 |  | 0.4±0.6 |  | |
| 127 | *Planctomyces* sp. (KF228168) | Planctomycetes | 91.3 | 6.0±6.2 | 1.8±1.4 | 1.7±0.8 |  | 2.8±1.4 |  | |
| 245 | *Prosthecobacter* sp. (JQ319004) | Verrucomicrobia | 85.2 | 3.8±2.8 | 3.0±1.4 | 2.0±0.2 |  | 1.7±0.7 |  | |
| 230 | *Acidobacterium* sp. (AM086241) | Acidobacteria | 96.4 | 1.3±0.8 | 2.6±2.3 | 1.3±02 |  | 1.4±0.3 |  | |
| 273 | *Beggiatoa* sp. (JN674459) | Proteobacteria | 84.7 | 4.3±1.4 | 0.8±0.7 | 2.3±1.2 |  | 2.0±0.9 |  | |
| 187 | *Actinomycetales* sp. (X68467) | Acidobacteria | 96.0 | 1.6±0.6 | 3.4±2.5 | 1.9±0.02 |  | 3.1±1.4 |  | |
| 232 | *Granulicella* sp. (AM887757) | Acidobacteria | 97.6 | 0.9±0.3 | 5.3±3.0 | 1.5±1.2 |  | 2.3±1.1 |  | |
| 281 | *Prosthecobacter* sp. (KF228173) | Verrucomicrobia | 85.0 | 4.0±3.6 | 1.7±1.1 | 1.3±0.5 |  | 2.2±0.7 |  | |
| 172 | *Actinomycetale*s sp. (X68466) | Acidobacteria | 94.9 | 3.1±1.7 | 1.0±0.1 | 1.2±0.7 |  | 5.8±3.9 |  | |
| 299 | *Granulicella* sp. (AM887757) | Acidobacteria | 93.1 | 2.8±2.0 | 0.7±0.03 | 3.3±0.9 |  | 2.9±0.2 |  | |
| 310 | *Methylocella* sp. (FN870334) | Proteobacteria | 98.4 | 2.8±2.1 | 0.1±0.2 | 2.0±1.0 |  | 1.3±0.2 |  | |
| 126 | *Flavobacteria* sp. (AF491884) | Bacteroidetes | 97.6 | 1.3±1.5 | 3.4±2.2 | 1.2±0.5 |  | 5.6±2.1 |  | |
| 308 | *Imtechium* sp. (AY544767) | Proteobacteria | 100.0 | 0.05±0.09 | 3.7±4.3 | 1.1±0.1 |  | 1.4±1.0 |  | |
| 311 | *Desulfomonile* sp. (AM086646) | Proteobacteria | 90.1 | 3.7±2.0 | 0.2±0.2 | 0.5±0.6 |  | 3.8±0.5 |  | |
| 113 | *Azospirillum* sp. (GQ181133) | Proteobacteria | 93.3 | 4.6±3.9 | 0.2±0.1 | 4.0±2.5 |  | 1.7±0.5 |  | |
| 430 | *Acidobacterium* sp. (AB298536) | Acidobacteria | 92.3 | 2.2±3.4 | 0.7±0.9 | 4.1±3.4 |  | 1.8±2.6 |  | |
| 545 | *Nitrosospira* sp. (AF386756) | Proteobacteria | 96.4 | 0.3±0.5 | 2.5±4.3 | 0.6±0.4 |  | 1.9±0.7 |  | |
| 201 | *Flavisolibacter* sp. (HM130561) | Bacteroidetes | 96.0 | 0.3±0.4 | 1.9±3.0 | 2.2±1.2 |  | 0.6±0.1 |  | |
| 353 | *Chryseolinea* sp. | Bacteroidetes | 95.3 | 0.03±0.06 | 2.0±3.5 | 1.4±1.0 |  | 1.3±0.3 |  | |
| 418 | *Opitutus* sp. (GQ902884) | Verrucomicrobia | 100.0 | 0±0 | 5.6±9.8 | 2.3±1.3 |  | 0.3±0.3 |  | |
| 437 | *Nitrosospira* sp. (KC477402) | Proteobacteria | 95.7 | 0.1±0.2 | 4.3±7.4 | 4.5±5.3 |  | 0.8±0.6 |  | |
| 486 | *Methyloversatilis* sp. (KC860260) | Proteobacteria | 93.7 | 0.05±0.09 | 2.8±4.9 | 0.4±0.2 |  | 1.1±0.5 |  | |
| 639 | *Verrucomicrobium* sp. (GQ304751) | Verrucomicrobia | 87.6 | 0.7±1.3 | 2.0±3.5 | 1.7±2.5 |  | 3.1±2.7 |  | |
| 167 | *Sphingomonas* sp. (KC493207) | Proteobacteria | 100.0 | 0±0 | 3.0±4.6 | 7.8±11.5 |  | 2.1±0.6 |  | |
| 193 | *Hymenobacter* sp. (JX949242) | Bacteroidetes | 98.8 | 0±0 | 7.7±13.4 | 2.9±3.9 |  | 1.9±1.4 |  | |
| 224 | *Sphingomonas* sp. (KC987002) | Proteobacteria | 100.0 | 0±0 | 11.6±20.1 | 0.1±0.1 |  | 1.9±2.2 |  | |
| 537 | *Hymenobacter* sp. (KC763792) | Bacteroidetes | 99.2 | 0.03±0.05 | 1.6±2.9 | 2.7±1.9 |  | 0.4±0.2 |  | |
| 995 | *Opitutus* sp. | Verrucomicrobia | 95.7 | 0±0 | 1.7±2.9 | 0.8±0.8 |  | 2.4±2.4 |  | |
|  |  |  |  |  |  |  |  |  |  | |
|  |  |  |  |  |  |  |  |  |  | |
|  |  |  |  |  |  |  |  |  |  | |
|  |  |  |  |  |  |  |  |  |  | |
|  |  |  |  |  |  |  |  |  |  | |
|  |  |  |  |  |  |  |  |  |  | |
|  |  |  |  |  |  |  |  |  |  | |
|  |  |  |  |  |  |  |  |  |  | |
|  |  |  |  |  |  |  |  |  |  | |
|  |  |  |  |  |  |  |  |  |  | |
|  |  |  |  |  |  |  |  |  |  | |
|  |  |  |  |  |  |  |  |  |  | |
|  |  |  |  |  |  |  |  |  |  | |
|  |  |  |  |  |  |  |  |  |  | |
|  |  |  |  |  |  |  |  |  |  | |
|  |  |  |  |  |  |  |  |  |  | |
|  |  |  |  |  |  |  |  |  |  | |
|  |  |  |  |  |  |  |  |  |  | |
|  |  |  |  |  |  |  |  |  |  | |
|  |  |  |  |  |  |  |  |  |  | |
|  |  |  |  |  |  |  |  |  |  | |
